# The rapid generation of isothiocyanates in flow

**DOI:** 10.3762/bjoc.9.184

**Published:** 2013-08-08

**Authors:** Marcus Baumann, Ian R Baxendale

**Affiliations:** 1 Department of Chemistry, University of Durham, South Road, Durham DH1 3LE, United Kingdom

**Keywords:** chloroxime, dipolar cycloaddition, flow chemistry, flow synthesis, immobilised reagents, isothiocyanate, nitrile oxide

## Abstract

Isothiocyanates are versatile starting materials for a wide range of chemical reactions. However, their high nucleophilic susceptibility means they are best prepared and used immediately. We report here on a flow platform for the fast and efficient formation of isothiocyanates by the direct conversion of easily prepared chloroximes. To expedite this chemistry a flow insert cartridge containing two immobilised reagents is used to affect the chemical transformation which typically eliminates the requirements for any conventional work-up or purification of the reaction stream.

## Introduction

Flow based chemical synthesis is playing an increasingly pivotal role within the chemical sciences as it facilitates a more versatile and responsive workflow. It encompasses many aspects of synthesis from the rapid and on-demand preparation of important building blocks to the development of multi-step sequences leading to advanced chemical structures and more direct scaling of bulk producing reactions [1–10]. Consequently, the chemical literature is growing at a precipitous rate with numerous examples of key chemical transformations having been further optimised or improved upon when conducted within a pseudo-/continuous flow process [11–12]. From a synthesis perspective the majority of these endeavours have been directed at enhancing specific reaction safety profiles, identifying new reaction sequences or generating improvements to well-known yet cumbersome transformations [13–16]. Indeed, flow chemistry is moving from an academic curiosity to become a common tool in many synthesis laboratories paralleling the emergence and adoption of another enabling technology, the microwave reactor [17].

**Scheme 1:**
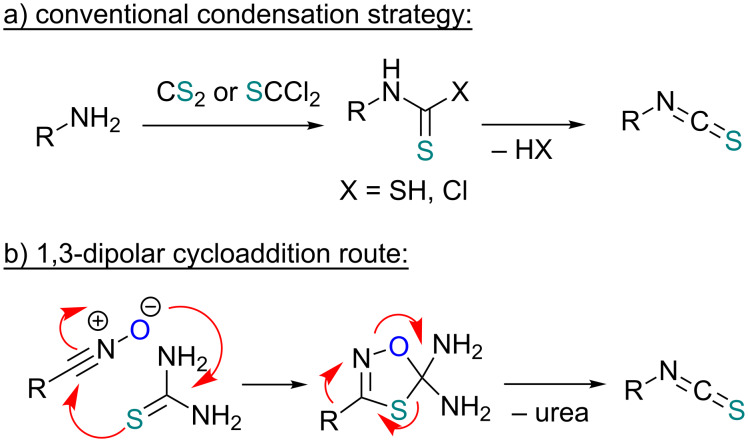
Strategies towards isothiocyanates.

An area which has benefited significantly from the many recent developments in our understanding of flow chemistry is the synthesis of reactive precursors and handling of sensitive intermediates [18–23]. However, despite the breadth of chemistries already explored there are certain functional transformations notably absent, one particular class of important building blocks which has received less attention are isothiocyanates. These species are widely utilised as valuable starting materials for many thiourea-based organocatalysts [24–26], numerous heterocyclic entities [27–29] as well as important entry points towards other key functional groups such as isocyanides [30–31], guanidines [32–33] and thiosemicarbazides [34]. Due to the limited commercial availability of diversely functionalised isothiocyanates chemists normally pursue a de novo synthesis, which most commonly involves the condensation of an amine with thiophosgene or carbon disulfide [35–36], both reagents causing safety concerns due to the formation of toxic, malodorous and/or extremely corrosive byproducts (Scheme 1a). An underutilised alternative sequence is the 1,3-dipolar cycloaddition reaction between a nitrile oxide and a thiourea compound which initially generates an unstable 1,4,2-oxathiazoline intermediate that readily rearranges into urea and eliminates the desired isothiocyanate product [37–38] (Scheme 1b).

Whilst this approach appears on initial inspection to be very attractive it is somewhat hampered by the requirement to access the reactive nitrile oxide species, which once formed is prone to undergo fast dimerisation leading to furoxan byproducts (see Table 1) [39]. In fact this facile dimerisation reaction is regularly reported as troublesome in many reactions that progress through nitrile oxide intermediates. As a consequence a number of strategies to minimise this side reaction have been attempted. These include the use of highly diluted reaction mixtures, the use of large excesses of the corresponding dipolarophile partner, as well as the slow addition of reagents to create limiting concentrations of the active 1,3-dipole. Despite these efforts, the side reaction is still seen and there normally remains the requirement for time consuming purifications such as column chromatography in order to isolate pure products.

Immobilised reagents have shown great promise as enabling technologies when incorporated in flow reactors to aid in the processing, work-up and purification of reaction sequences [40–44]. In addition they have been successfully utilised in order to render dipolar cycloaddition reactions involving azomethine ylides [45–46] as well as nitrile oxides [47–48] more practical for generating important heterocyclic scaffolds such as pyrrolidines, isoxazolines and their derivatives [49–52]. We therefore considered that it should be possible to develop a mild and practical flow-based process to form isothiocyanates from the corresponding reactive nitrile oxide intermediates.

**Scheme 2:**
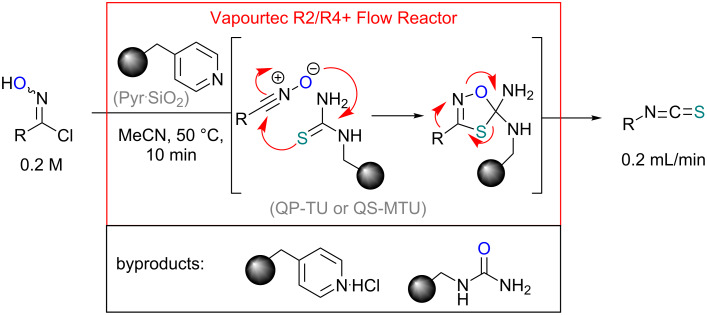
Flow approach towards isothiocyanates.

Our strategy makes use of two immobilised reagents (a weak base and a functionalised thiourea) placed as a 1:1-mixture within a single glass reactor cartridge (Scheme 2). The immobilised base affects the in situ formation of the reactive nitrile oxide from the chloroxime which is immediately captured by the local tethered thiocarbonyl dipolarophile. Consequently, this approach significantly minimises the formation of the furoxan byproduct by creating a scenario of pseudo high dilution of the nitrile oxide whilst allowing a concurrent high local concentration of the dipolarophile. Additionally the immobilised supports act as excellent sequestering agents for trapping even the small quantities of polar impurities within the matrix thus allowing for the isolation of the desired isothiocyanate product in high purity after only simple solvent removal.

## Results and Discussion

Initially a set of batch experiments was performed using (4-bromophenyl)chloroxime **1a** as the substrate, which was efficiently prepared on gram scale from the corresponding benzaldehyde via oxime formation and subsequent NCS-mediated chlorination [53]. Screening different solvents (MeCN, acetone, EtOAc, MeOH and iPrOH) and immobilised thiourea species (QP-TU [54] and QS-MTU [55]) showed that the desired transformation can be effected with either immobilised thiourea source and furthermore tolerates a wide range of the solvents, with MeCN being identified as the best option. The choice of base was found to be of particular importance given that solution phase bases tested (NEt_3_ or DBU even using slow addition) immediately generated substantial quantities of a white precipitate, which was later identified as the undesired furoxan byproduct (Table 1). In order to evaluate conditions to minimise this side reaction we studied several immobilised bases including QP-DMA (benzyldimethylamine resin) and SiO_2_-pyr. The latter allowed for the isolation of the desired isothiocyanate in high yield and with very little contamination by the furoxan byproduct.

**Table 1:** Screening of different bases for the formation and conversion of nitrile oxides in batch:

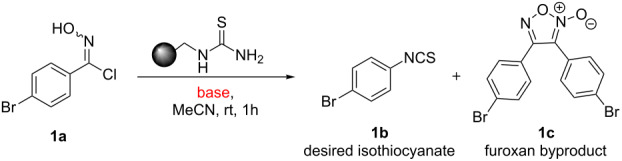			

Entry	Base	Ratio isothiocyanate/furoxan	Comments

1	NEt_3_ (0.2 M in MeCN)	1:1	dropwise addition of base
2	DBU (0.2 M in MeCN)	1:1	dropwise addition of base
3	QP-DMA	2.2:1	portionwise addition of base
4	SiO_2_-pyr	>4:1	portionwise addition of base

We translated the outcome of these preliminary results into a flow protocol using a commercially available Vapourtec R series flow reactor [56] which was operated with MeCN as the system solvent. Stock solutions of the chloroxime starting materials were prepared in the same solvent (0.25–0.5 M) and injected into a sample loop (2–10 mL size). Both the solid supported base (SiO_2_-pyr) and immobilised thiourea species (QP-TU or QS-MTU) were blended (by shaking), filled into an glass column (10 cm length, 6.6 mm i.d., 1:1 ratio, 1.2 equiv each species) and the column inserted into a glass heating jacket which was maintained at 50 °C. The flow stream of the substrate was subsequently directed through the heated reactor column at a flow rate of 0.2 mL/min equating to an average residence time of 10 minutes. After passing a 100 psi backpressure regulator the typically colourless reaction mixture was collected yielding the product after evaporation of the solvent. ^1^H NMR experiments were used to determine the product composition and purity and pleasingly revealed complete conversion of the substrates into the desired isothiocyanate product in high yield and purity as summarised in Table 2.

**Table 2:** Isothiocyanates prepared in flow.

Entry	Starting material	Product	Isolated yield [%]	Scale [mmol]

**1**	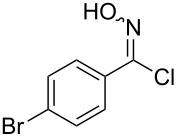	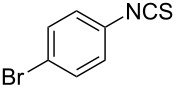 **1b**	93^a^ 84^a^	1.0 5.0
**2**	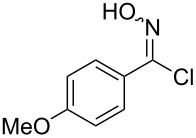	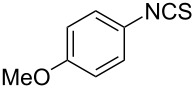 **2b**	89^a^	1.0
**3**	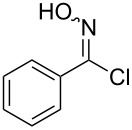	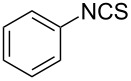 **3b**	91^a^	1.0
**4**	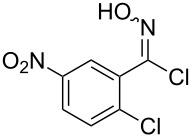	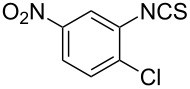 **4b**	78^a^	0.7
**5**	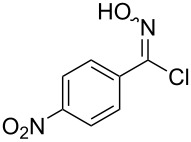	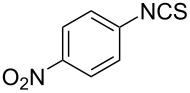 **5b**	76^a^	0.5
**6**	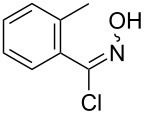	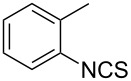 **6b**	94^a^	1.0
**7**	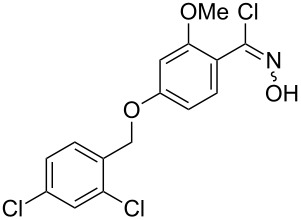	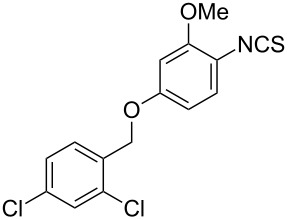 **7b**	91^a^	0.5
**8**	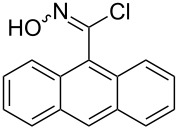	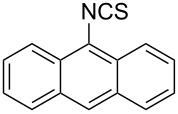 **8b**	85^a^	1.0
**9**	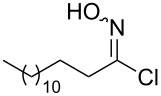	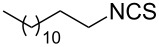 **9b**	89^a^	0.8
**10**	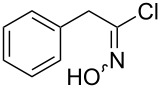	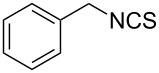 **10b**	84^a^	0.8
**11**	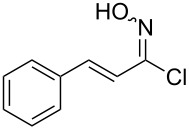	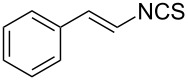 **11b**	81^a^	0.8
**12**	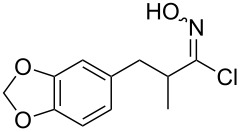	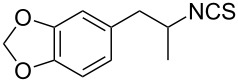 **12b**	87^a^	1.0
**13**	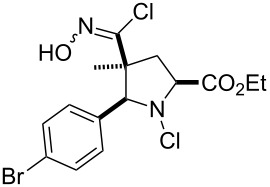	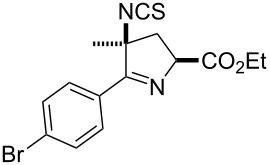 **13b**	73^b,c^	0.5
**14**	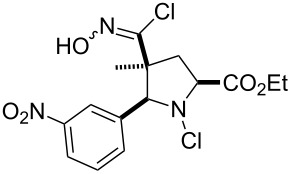	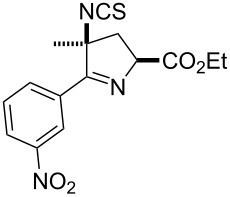 **14b**	71^b,c^	0.5
**15**	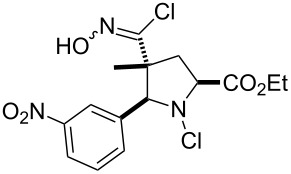	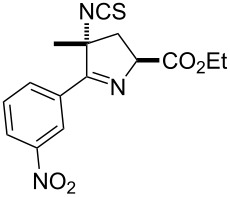 **15b**	76^b,c^	0.75

^a^Purity > 90% by ^1^H NMR. ^b^Purified by SiO_2_ column chromatography. ^c^2.5 equiv of SiO_2_-pyridine used.

The reactions demonstrate a high tolerance for both electron poor and electron rich aromatic substrates (Table 2, entries 1–8) which all delivered the desired isothiocyanates reliably in high yield and purity. Furthermore, aliphatic chloroximes were found to work equally well, efficiently generating the corresponding isothiocyanate products again in high yields and purities (Table 2, entries 9–12). When using single diastereoisomers of α-chiral chloroximes the desired isothiocyanate product was correspondingly isolated as a single isomer (by ^1^H NMR) suggesting a concerted reaction pathway to be in operation. Importantly, more complex substrates (i.e. *N*-chloropyrrolidines, Table 2, entries 13–15) can be subjected to the reaction conditions in order to not only install the desired isothiocyanate functionality but through concomitant elimination affect the formation of a cyclic imine, which is very attractive as it allows subsequent diversification of these polyfunctionalised heterocyclic building blocks. Overall, this exemplifies how the presented methodology can enable the rapid construction of scaffolds with unprecedented substitution patterns, which hold great interest due to their potential as highly decorated entities.

The most noteworthy feature of this flow protocol is its efficiency and simplicity allowing the desired product to be isolated following only solvent removal. The incomplete recovery of material in these reactions can be accounted for by small amounts of the furoxan dimer which forms yet is trapped within the reactor column, thus evading its tedious removal via subsequent column chromatography. In addition our study demonstrates that thiourea – either immobilised on a polystyrene-type support (QP-TU) or on silica gel (QS-MTU) – serves as an efficient source of a sulfur atom. Given the higher loading of QP-TU (~5.5 mmol/g) compared to QS-MTU (~1.5 mmol/g) as well as its lower cost, QP-TU might be considered the more economical option, however, with the primary focus of this study being the small scale generation of valuable novel isothiocyanate products, this would only become relevant at larger scales (>100 g).

## Conclusion

In conclusion, we have demonstrated an efficient and mild flow protocol for the synthesis of isothiocyanates from readily available chloroximes exploiting several immobilised reagents. This approach allows for the rapid access to various isothiocyanate building blocks avoiding time consuming purifications. Our study expands on the traditional use of immobilised thiourea species as simple metal scavengers and will likely lead to their further application as a convenient source of sulfur.

## Supporting Information

File 1 Experimental part.
