# Supplementary material for: The rapid generation of isothiocyanates in flow
Source: Beilstein J Org Chem. 2013 Aug 8;9:1613–9. doi: 10.3762/bjoc.9.184 (PMC3778409; doi:10.3762/bjoc.9.184)
Supplement: File 1 — Experimental part. [file Beilstein_J_Org_Chem-09-1613-s001.pdf]

Supporting Information

for

The rapid generation of isothiocyanates in flow

Marcus Baumann and Ian R. Baxendale*

Address: Department of Chemistry, University of Durham, South Road, Durham DH1 3LE, United Kingdom

Email: Ian R. Baxendale - i.r.baxendale@durham.ac.uk

* Corresponding author

Experimental part

Experimental

General experimental procedures: ^{1}H and ^{13}C NMR spectra were recorded on either Bruker-Avance 400 or Varian VNMRS-600 instruments using $CDCl_3$ as internal reference. HSQC, HMBC and NOESY experiments were used in aiding the structural assignment. IR spectra were recorded neat on a Perkin Elmer RX1 spectrometer (ATR sampling) with the intensities of the characteristic signals being reported as weak (w, <20% of tallest signal), medium (m, 21–70% of tallest signal) and strong (s, >70% of tallest signal). Low and high resolution mass spectrometry was performed using the indicated techniques on either a Thermo-Finnigan Trace GC–MS or Waters LCT Premier XE and Waters TQD instruments equipped with Acquity UPLC and a lock-mass electrospray ion source.

General flow procedure for the conversion of chloroximes into isothiocyanates:

A solution of a chloroxime substrate was prepared in 2 mL MeCN (0.25–0.5 M) and injected into a sample loop mounted on the R2-flow unit. This solution was subsequently pumped at a flow rate of 0.2 mL/min into a heated Omnifit glass column (50 °C, 10 cm length, 6.6 mm i.d.) placed in a glass jacket on the R4-flow unit. This glass column was filled with a mixture of the immobilised base (SiO_2-pyridine, 1.2 equiv [or 2.5 equiv in case of entries 13, 14, 15 in Table 2]) and the immobilised thiourea (QP-TU or QS-MTU, 1.2 equiv). After passing this reactor column the reaction mixture was collected, concentrated by evaporation of the solvent and analysed by ^{1}H NMR spectroscopy. If the product purity was below 90% column chromatography was performed as indicated below (10% EtOAc/90% hexanes).

4-Bromophenylisothiocyanate (1b):

Isolated as white solid after evaporation of the solvent (198 mg, 93%). ^{1}H NMR ($CDCl_3$, 400 MHz) δ 7.46 (2H, d, *J* = 8.0 Hz), 7.08 (2H, d, *J* = 8.0 Hz); ^{13}C NMR ($CDCl_3$, 100 MHz) δ 137.0 (C), 132.8 (2CH), 130.5 (C), 127.2 (2CH), 120.8 (C); IR (neat) v/cm^{-1} 3082.4 (w), 2168.4 (m), 2067.7 (s), 1579.8 (m), 1477.6 (s), 1400.0 (s), 1066.7 (m), 923.0 (m), 816.6 (s); GC–MS: 212.8 (M^+); HRMS–ESI: calculated for C_7H_5BrNS: 213.9326, found: 213.9308 (M + H^+).

Spectroscopic data are in accordance with published data from supplier [1].

4-Methoxyphenylisothiocyanate (2b):

Isolated as white solid after evaporation of the solvent (146 mg, 89%). ^{1}H NMR ($CDCl_3$, 600 MHz) δ 7.15 (2H, d, *J* = 9.0 Hz), 6.84 (2H, d, *J* = 9.0 Hz), 3.80 (3H, s); ^{13}C NMR ($CDCl_3$, 150 MHz) δ 158.6 (C), 133.9 (C), 126.9 (2CH), 123.6 (C), 114.8 (2CH), 55.6 (CH_3); IR (neat) v/cm^{-1} 2835.1 (w), 2068.9 (s), 1602.0 (m), 1501.3 (s), 1461.9 (m), 1104.9 (m), 1031.3 (m), 926.8 (m), 825.7 (s); ASAP–MS: 165.0 (M + H^+); HRMS–AP$^+$: calculated for C_8H_7NOS: 165.0248, found: 165.0249 (M^+).

Spectroscopic data are in accordance with published data from supplier [1].

Phenylisothiocyanate (3b):

Isolated as colourless oil after evaporation of the solvent (122 mg, 91%). ^{1}H NMR ($CDCl_3$, 400 MHz) δ 7.27 (2H, t, *J* = 8.0 Hz), 7.20 (1H, m), 7.10-7.16 (2H, m); ^{13}C NMR ($CDCl_3$, 100 MHz) δ 135.3 (C), 131.3 (C), 129.6 (2CH), 127.3 (CH), 125.7 (2CH); IR (neat) v/cm^{-1} 2171.9 (m), 2050.5 (s), 1590.4 (s), 1489.0 (s), 1451.6 (w), 1069.9 (w),

925.0 (m), 904.8 (m), 746.5 (s), 682.1 (s); ASAP–MS: 136.0 (M + H^+); HRMS–AP^+: calculated for C_7H_5NS: 135.0143, found: 135.0147 (M^+).

Spectroscopic data are in accordance with published data from supplier [1].

2-Chloro-5-nitrophenylisothiocyanate (4b) [2]:

Isolated as white solid after evaporation of the solvent (117 mg, 78%). ^{1}H NMR ($CDCl_3$, 400 MHz) δ 8.09 (1H, s), 8.06 (1H, d, J = 8.0 Hz), 7.60 (1H, d, J = 8.0 Hz); ^{13}C NMR ($CDCl_3$, 100 MHz) δ 146.9 (C), 142.1 (C), 138.7 (C), 131.5 (C), 130.9 (CH), 122.2 (CH), 121.5 (CH); IR (neat) v/cm^{-1} 3095.7 (w), 2010.1 (s), 1519.6 (s), 1511.1 (s), 1463.8 (m), 1057.3 (m), 891.9 (s), 837.8 (s), 739.2 (s); ASAP–MS: 215.0 (M + H^+); HRMS–AP^+: calculated for $C_7H_3ClN_2O_2S$: 213.9604, found: 213.9591 (M^+).

4-Nitrophenylisothiocyanate (5b):

Isolated as white solid after evaporation of the solvent (68 mg, 76%). ^{1}H NMR ($CDCl_3$, 400 MHz) δ 8.24 (2H, d, J = 8.8 Hz), 7.35 (2H, d, J = 8.8 Hz); ^{13}C NMR ($CDCl_3$, 100 MHz) δ 145.8 (C), 140.4 (C), 137.9 (C), 126.4 (2CH), 125.3 (2CH); IR (neat) v/cm^{-1} 2101.0 (s), 1603.1 (w), 1584.7 (s), 1518.9 (s), 1485.7 (m), 1335.7 (s), 1105.9 (m), 850.4 (s), 745.9 (m); ASAP–MS: 180.0 (M^+); HRMS–AP^+: calculated for $C_7H_4N_2O_2S$: 179.9993, found: 179.9990 (M^+).

Spectroscopic data are in accordance with published data from supplier [1].

1-Isothiocyanato-2-methylbenzene (6b):

Isolated as colourless oil after evaporation of the solvent (140 mg, 94%). ^{1}H NMR ($CDCl_3$, 400 MHz) δ 7.13-7.24 (4H, m), 2.38 (3H, s); ^{13}C NMR ($CDCl_3$, 100 MHz) δ 135.3 (C), 135.0 (C), 130.7 (CH), 130.3 (C), 127.4 (CH), 126.9 (CH), 125.9 (CH), 18.4 (CH_3); IR (neat) v/cm^{-1} 2062.8 (s), 1598.2 (w), 1485.2 (m), 1459.8 (m), 1114.2 (w), 927.2 (s), 787.9 (m), 749.5 (s), 672.2 (m); ASAP–MS: 149.0 (M^+); HRMS–AP$^+$: calculated for C_8H_7NS: 149.0299, found: 149.0296 (M^+).

Spectroscopic data are in accordance with published data from supplier [1].

2,4-Dichloro-1-((4-isothiocyanato-3-methoxyphenoxy)methyl)benzene (7b):

Isolated as white solid after evaporation of the solvent (155 mg, 91%). ^{1}H NMR ($CDCl_3$, 400 MHz) δ 7.54 (1H, d, *J* = 2.0 Hz), 7.45 (1H, d, *J* = 7.6 Hz), 7.25 (1H, dd, *J* = 2.0, 8.0 Hz), 6.87 (1H, dd, *J* = 2.0, 8.4 Hz), 6.82 (1H, d, *J* = 8.4 Hz), 6.72 (1H, d, *J* = 2.0 Hz), 5.04 (2H, s), 3.88 (3H, s); ^{13}C NMR ($CDCl_3$, 100 MHz) δ 149.2 (C), 148.0 (C), 136.5 (C), 134.2 (C), 132.9 (C), 132.2 (C), 130.7 (CH), 129.2 (CH), 126.5 (CH), 123.6 (C), 119.4 (CH), 112.0 (CH), 111.7 (CH), 69.8 (CH_2), 56.2 (CH_3); IR (neat) v/cm^{-1} 2844.8 (w), 2126.0 (s), 1587.4 (m), 1505.3 (s), 1378.0 (m), 1260.0 (s), 1238.0 (s), 1216.8 (m), 1176.5 (s), 1127.8 (s), 1019.1 (s), 832.4 (s), 800.0 (m); ASAP–MS: 340.0 (M + H^+); HRMS–AP$^+$: calculated for $C_{15}H_{11}NO_2Cl_2S$: 338.9888, found: 338.9911 (M^+).

9-Isothiocyanatoanthracene (8b) [3]:

Isolated as yellow solid after evaporation of the solvent (200 mg, 85%). ^{1}H NMR ($CDCl_3$, 600 MHz) δ 8.37 (1H, s), 8.28 (2H, d, *J* = 9.0 Hz), 8.01 (2H, d, *J* = 9.0 Hz), 7.61 (2H, t, *J*

= 7.8 Hz), 7.53 (2H, t, *J* = 7.8 Hz); ^{13}C NMR ($CDCl_3$, 150 MHz) δ 137.7 (C), 131.6 (C), 129.0 (CH), 128.0 (C), 127.5 (CH), 126.7 (CH), 126.3 (CH), 123.2 (CH), 122.4 (C); IR (neat) v/cm^{-1} 3052.0 (w), 2114.2 (s), 1710.0 (w), 1673.8 (w), 1442.0 (w), 886.7 (m), 777.3 (m), 730.0 (s); ASAP–MS: 236.0 (M + H^+); HRMS–AP$^+$: calculated for $C_{15}H_9NS$: 235.0456, found: 235.0430 (M^+).

1-Isothiocyanatotridecane (9b):

Isolated as colourless oil after evaporation of the solvent (171 mg, 89%). ^{1}H NMR ($CDCl_3$, 400 MHz) δ 3.44 (2H, t, *J* = 6.4 Hz), 1.63 (2H, m), 1.15-1.40 (20H, m), 0.81 (3H, t, *J* = 6.8 Hz); ^{13}C NMR ($CDCl_3$, 100 MHz) δ 129.0 (C), 45.1 (CH_2), 31.9 (CH_2), 30.0 (CH_2), 29.7 (CH_2), 29.64 (CH_2), 29.61 (CH_2), 29.5 (CH_2), 29.4 (CH_2), 29.36 (CH_2), 28.8 (CH_2), 26.6 (CH_2), 22.7 (CH_2), 14.1 (CH_3); IR (neat) v/cm^{-1} 2921.3 (s), 2852.2 (m), 2084.8 (s), 1455.2 (m), 1345.3 (m), 721.0 (w); ASAP–MS: 242.2 (M + H^+); HRMS–AP$^+$: calculated for $C_{14}H_{28}NS$: 242.1942, found: 242.1948 (M + H^+).

Benzylisothiocyanate (10b):

Isolated as colourless oil after evaporation of the solvent (100 mg, 84%). ^{1}H NMR ($CDCl_3$, 400 MHz) δ 7.30-7.45 (5H, m), 4.72 (2H, s); ^{13}C NMR ($CDCl_3$, 100 MHz) δ 134.3 (C), 132.2 (C), 129.0 (2CH), 128.4 (CH), 126.9 (2CH); IR (neat) v/cm^{-1} 2166.1 (m), 2079.9 (s), 1495.8 (m), 1453.8 (m), 1438.1 (m), 1346.3 (m), 1028.0 (w), 696.3 (s); GC–MS: 148.9 (M^+).

Spectroscopic data are in accordance with published data from supplier [1].

(*E*)-(2-Isothiocyanatovinyl)benzene (11b) [4]:

Isolated as colourless oil after evaporation of the solvent (104 mg, 81%). ^{1}H NMR ($CDCl_3$, 400 MHz) δ 7.30-7.38 (5H, m), 6.67 (1H, d, *J* = 14.0 Hz), 5.58 (1H, d, *J* = 14.0 Hz); ^{13}C NMR ($CDCl_3$, 100 MHz) δ 134.1 (C), 133.7 (C), 131.6 (CH), 129.0 (CH), 128.9 (2CH), 126.3 (2CH), 115.4 (CH); IR (neat) v/cm^{-1} 2099.6 (s), 1625.5 (w), 1447.4 (w), 932.8 (m), 746.3 (m), 689.8 (m); ASAP–MS: 162.0 (M + H^+); HRMS–AP$^+$: calculated for C_9H_7NS: 161.0299, found: 161.0293 (M^+).

5-(2-Isothiocyanatopropyl)benzo[*d*][1,3]dioxole (12b):

Isolated as white solid after evaporation of the solvent (192 mg, 87%). ^{1}H NMR ($CDCl_3$, 600 MHz) δ 6.77 (1H, d, *J* = 7.8 Hz), 6.68 (1H, s), 6.65 (1H, d, *J* = 7.8 Hz), 5.95 (2H, s), 3.90 (1H, m), 2.75-2.86 (2H, m), 1.36 (3H, d, *J* = 6.6 Hz); ^{13}C NMR ($CDCl_3$, 150 MHz) δ 147.8 (C), 146.7 (C), 131.5 (C), 130.3 (C), 122.5 (CH), 109.5 (CH), 108.4 (CH), 101.0 (CH2), 55.3 (CH), 43.5 (CH_2), 21.3 (CH_3); IR (neat) v/cm^{-1} 2886.2 (w), 2088.0 (s), 1501.8 (s), 1487.9 (s), 1442.0 (s), 1332.1 (m), 1246.4 (s), 1190.1 (m), 1100.3 (m), 1035.8 (s), 926.7 (s), 807.6 (m); ASAP–MS: 222.0 (M + H^+); HRMS–AP$^+$: calculated for $C_{11}H_{11}NO_2S$: 221.0511, found: 221.0519 (M^+).

rac-(2*S*,4*S*)-Ethyl 5-(4-bromophenyl)-4-isothiocyanato-4-methyl-3,4-dihydro-2*H*-pyrrole-2-carboxylate (13b):

Isolated after column chromatography (10% EtOAc, 90% hexanes) as colourless oil (134 mg, 73%). ^{1}H NMR ($CDCl_3$, 600 MHz) δ 7.89 (2H, d, *J* = 9.0 Hz), 7.59 (2H, d, *J* = 9.0 Hz), 4.67 (1H, app. t, *J* = 7.8 Hz), 4.28 (2H, q, *J* = 7.2 Hz), 2.73 (1H, dd, *J* = 8.4,

13.2 Hz), 2.65 (1H, dd, *J* = 7.2, 13.2 Hz), 1.71 (3H, s), 1.33 (3H, t, *J* = 7.2 Hz); ^{13}C NMR ($CDCl_3$, 150 MHz) δ 172.1 (C), 170.6 (C), 136.6 (C), 131.9 (2CH), 129.9 (2CH), 129.4 (C), 126.3 (C), 71.0 (C), 69.8 (CH), 61.8 (CH_2), 44.9 (CH_2), 25.9 (CH_3), 14.2 (CH_3); IR (neat) v/cm^{-1} 2980.9 (w), 2055.9 (s), 1735.9 (s), 1588.0 (m), 1487.2 (m), 1271.1 (m), 1192.5 (s), 1106.9 (m), 1071.8 (m), 1010.1 (m), 833.3 (m); LC–MS: 368.9 (M + H^+); HRMS–AP$^+$: calculated for $C_{15}H_{16}O_2BrN_2S$: 367.0110, found: 367.0117.

rac-(2*S*,4*S*)-Ethyl 4-isothiocyanato-4-methyl-5-(3-nitrophenyl)-3,4-dihydro-2*H*-pyrrole-2-carboxylate (14b):

Isolated after purification by column chromatography (10% EtOAc, 90% hexanes) as yellow oil (117 mg, 71%). ^{1}H NMR ($CDCl_3$, 400 MHz) δ 8.88 (1H, t, *J* = 2.4 Hz), 8.33-8.38 (2H, m), 7.67 (1H, t, *J* = 8.0 Hz), 4.75 (1H, app. t, *J* = 8.0 Hz), 4.31 (2H, q, *J* = 7.2 Hz), 2.80 (1H, dd, *J* = 8.4, 13.2 Hz), 2.71 (1H, dd, *J* = 7.6, 13.2 Hz), 1.75 (3H, s), 1.35 (3H, t, J = 7.2 Hz); ^{13}C NMR ($CDCl_3$, 100 MHz) δ 171.3 (C), 170.4 (C), 148.5 (C), 138.1 (C), 134.0 (CH), 132.4 (C), 129.9 (CH), 125.9 (CH), 123.6 (CH), 71.2 (C), 70.0 (CH), 62.0 (CH_2), 44.9 (CH_2), 25.9 (CH_3), 14.2 (CH_3); IR (neat) v/cm^{-1} 2981.7 (w), 2064.0 (s), 1710.4 (s), 1529.9 (s), 1446.1 (m), 1348.1 (s), 1186.6 (m), 1096.4 (m), 911.3 (m), 734.7 (m); ASAP–MS: 334.1 (M + H^+); HRMS–AP$^+$: calculated for $C_{15}H_{16}O_4N_3S$: calculated for $C_{15}H_{16}O_4N_3S$: 334.0862, found: 334.0881 (M + H^+).

rac-(2*S*,4*R*)-Ethyl 4-isothiocyanato-4-methyl-5-(3-nitrophenyl)-3,4-dihydro-2*H*-pyrrole-2-carboxylate (15b):

Isolated after column chromatography (10% EtOAc, 90% hexanes) as yellow oil (188 mg, 76%). ^{1}H NMR ($CDCl_3$, 600 MHz) δ 8.83 (1H, s), 8.36 (1H, d, *J* = 8.4 Hz), 8.28 (1H, d, *J* = 8.4 Hz), 7.67 (1H, t, *J* = 8.4 Hz), 5.05 (1H, dd, *J* = 5.4, 8.4 Hz), 4.28 (2H, q, *J* = 7.2 Hz), 2.85 (1H, dd, *J* = 8.4, 13.2 Hz), 2.60 (1H, dd, *J* = 5.4, 13.2 Hz), 1.82 (3H, s), 1.35 (3H, t, *J* = 7.2 Hz); ^{13}C NMR ($CDCl_3$, 150 MHz) δ 171.3 (C), 170.8 (C), 148.4 (C). 138.2 (C), 134.0 (CH), 132.5 (C), 129.8 (CH), 125.7 (CH), 123.6 (CH), 77.3 (C), 71.0 (CH), 62.0 (CH_2), 44.5 (CH_2), 27.1 (CH_3), 14.1 (CH_3); IR (neat) v/cm^{-1} 2036.2 (s), 1737.8 (s), 1531.1 (s), 1349.4 (s), 1190.0 (m), 1094.2 (m), 911.9 (m), 732.0 (s); ASAP–MS: 334.1 (M + H^+); HRMS–AP$^+$: calculated for $C_{15}H_{16}O_4N_3S$: 334.0862, found: 334.0872 (M + H^+).

References:

1 See the corresponding compound at www.sigmaaldrich.com

2 Khadse, B.G.; Shah, M.H.; Deliwala, C.V. *Bulletin of Haffkine Institute* **1975**, *3*, 27–32.

3 Janovec, L.; Suchar, G.; Imrich, J.; Kristian, P.; Sasinkova, V.; Alföldi, J.; Sedlak, E. *Collect. Czech. Chem. Commun*. **2002**, *67*, 665–678.

4 Kniezo, L.; Kristian, P. *Chemicke Zvesti* **1974**, *28*, 848–853.
